# Interaction between DRD2 and AKT1 genetic variations on risk of psychosis in cannabis users: a case–control study

**DOI:** 10.1038/npjschz.2015.25

**Published:** 2015-07-01

**Authors:** Marco Colizzi, Conrad Iyegbe, John Powell, Giuseppe Blasi, Alessandro Bertolino, Robin M Murray, Marta Di Forti

**Affiliations:** 1 Group of Psychiatric Neuroscience, Department of Basic Medical Sciences, Neuroscience and Sense Organs, University of Bari ‘Aldo Moro’, Bari, Italy; 2 Department of Psychosis Studies, Institute of Psychiatry, Psychology and Neuroscience, King’s College London, London, UK; 3 Department of Neuroscience, Institute of Psychiatry, Psychology and Neuroscience, King’s College London, London, UK; 4 pRED, NORD DTA, F. Hoffman-La Roche Ltd., Basel, Switzerland

## Introduction

Genetic factors may explain the differences in individual sensitivity to the psychosis-inducing effects of cannabis.^1,2^ In view of the converging data from candidate gene and genome-wide association studies that the D2-AKT1 signaling pathway is relevant for the pathophysiology and outcome of schizophrenia,^3^ and on the basis of previous association between cannabis-related psychosis and both *DRD2* (rs1076560)^1^ and *AKT1* (rs2494732),^2^ we hypothesized that these polymorphisms interact in increasing the risk of psychosis in cannabis users. We expected the genetic pathway × cannabis use interaction model to better predict the individual’s odds of psychotic disorder than the single candidate gene×cannabis use interaction model.

## Materials and Methods

Participants were recruited as part of the Genetic and Psychosis project (GAP),^2^ a case–control study, carried out at the Institute of Psychiatry, London. All patients presenting to the Adult services (18–65 years) of the South London and Maudsley Mental Health NHS Foundation Trust, between December 2005 and October 2010, with their first episode of psychosis, were recruited into the study. Over the same time frame, from the area served by the mental health units, we recruited a sample of control subjects, aged 18–65 years, representing the local population in terms of ethnicity and other main sociodemographics according to the appropriate census data (www.statistics.gov.uk/census). Using the same methodology as in ref. 2, only patients with a diagnosis of nonorganic psychosis (International Classification of Diseases, 10: F20–F29 and F30–F33) were included, and control subjects were excluded if they met criteria for a psychotic disorder or if they reported a previous diagnosis of psychotic illness. For a more detailed description of the GAP study methods, (see refs 1, 2).

The data presented here are based on the subset of the whole GAP sample (450 participants (222 first-episode psychosis patients and 228 healthy individuals) with complete information on the following: (i) sociodemographics (age, gender, and self-reported ethnicity); (ii) lifetime use of cannabis, stimulants, tobacco, and alcohol; and (iii) *DRD2* rs1076560 and *AKT1* rs2494732 genotypes. To confirm self-report of ethnicity, genetic ancestry was derived using a panel of 57 ancestry-informative genetic markers, as performed previously.^2^ These were genotyped using iPLEX technology developed for the MassArray platform (Sequenom, San Diego, CA, USA). Ancestry scores were derived using the program Structure to implement a model-based (Markov Chain Monte Carlo) clustering algorithm. Having determined the best solution for K (the probable true number of underlying genetic groups) in initial analyses, individuals who scored between 96 and 100% for genetic cluster membership were used to create a three-way ancestral axis on the basis of Black African (*N*=81), European Caucasian (*N*=118), and Asian (*N*=16) ancestry. These reference groups were used to index genetic ancestry for the remaining sample. Further information on the makeup of the marker panel as well as a figure reporting plots of three-way ancestral axis on the basis of Black African, European Caucasian, and Other are available on request. Ninety percent (*N*=407) of participants had information on both self-reported ethnicity and ancestry markers. The level of overall agreement between self-reported and genetic ethnicities (96%) was reassuringly high.

Multivariable logistic regressions were used to evaluate the main and interaction effects between two measures of cannabis exposure (lifetime use and frequency of use) and *DRD2* rs1076560*/AKT1* rs2494732 (individuals carrying one or more of each of the two ‘risk’ alleles (*DRD2* T *and AKT1* C); individuals carrying one or more ‘risk’ alleles of only one of the genes (*DRD2* T *or AKT1* C); individuals carrying no ‘risk’ alleles) on presence of a psychotic disorder, after adjusting for potential confounders (sociodemographics and other drug use). The interaction model examined the probability of having a psychotic disorder among cannabis users carrying ‘risk’ allele(s) from both (*DRD2* T carriers/*AKT1* C carriers) or only one of the genes (*DRD2* T carriers/*AKT1* TT and *DRD2* GG/*AKT1* C carriers) compared with *DRD2* GG/*AKT1* TT subjects. Odds ratio (OR) of psychosis among cannabis-naive subjects carrying ‘risk’ allele(s) from both or only one of the genes were also calculated from the estimates provided by the model.

The study was granted ethical approval by the South London and Maudsley and Institute of Psychiatry Local Research Ethics Committee. All cases and control subjects who were included gave informed written consent, signing the consent document, to the publication of data originating from the study.

## Results

**Figure 1:**
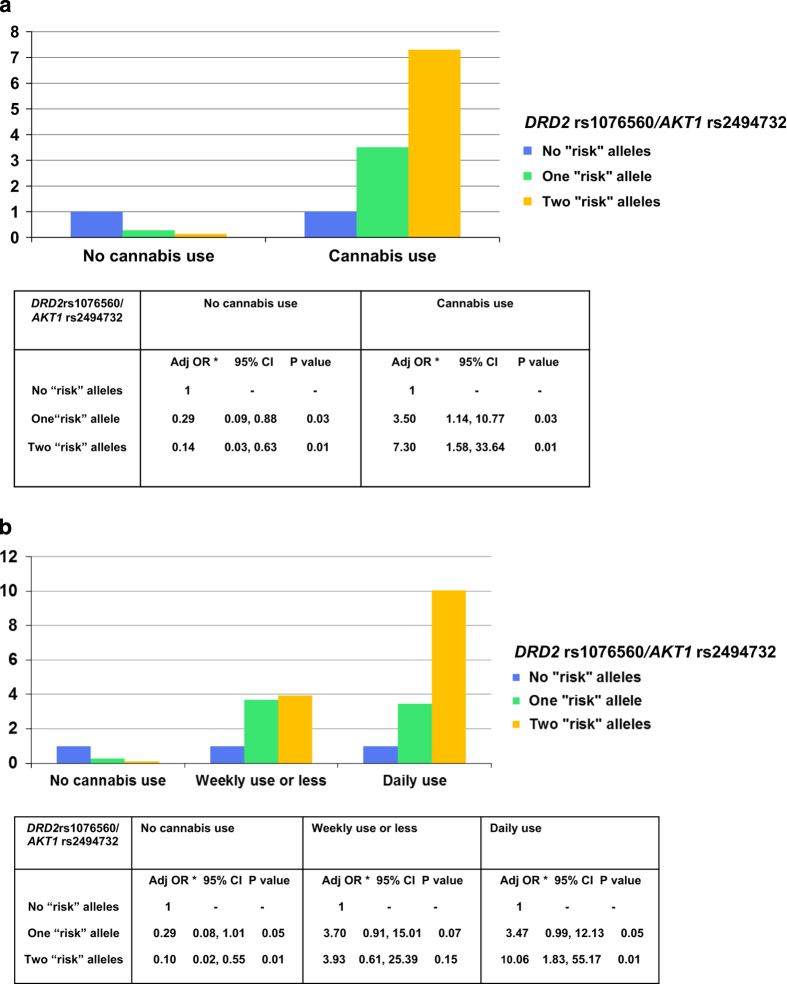
(**a**) Interaction between *DRD2* rs1076560*/AKT1* rs2494732 and lifetime cannabis use on psychosis risk. (**b**) Interaction between *DRD2* rs1076560*/AKT1* rs2494732 and lifetime frequency of cannabis use on psychosis risk. *Adjusted for gender, age, ethnicity, nicotine dependence, stimulants use, and harmful drinking behavior.

First-episode psychosis and control subjects differed significantly for some demographic characteristics and in their patterns of drug use. However, the *DRD2/AKT1* genotype was not associated with any sociodemographic variable or cannabis use (all *P*>0.1; Table 1). A multivariable logistic regression adjusting for the modeled potential confounders showed a significant interaction between *DRD2* rs1076560*/AKT1* rs2494732 genotypes and lifetime cannabis use on probability of suffering from a psychotic disorder. The analysis showed an increasing probability of suffering from a psychotic disorder in cannabis users depending on *DRD2/AKT1* (*N*=450, likelihood ratio test=7.66; *P*=0.022). When compared with the no ‘risk’ allele group, cannabis users carrying ‘risk’ allele(s) from only one (OR=3.50; 95% confidence interval: 1.14, 10.77) or both the genes (OR=7.30; 95% confidence interval: 1.58, 33.64) showed increased odds of having psychotic disorder. On the contrary, among those who had never used cannabis, carrying ‘risk’ allele(s) from only one (OR=0.29; 95% confidence interval: 0.09, 0.88) or both the genes (OR=0.14; 95% confidence interval: 0.03, 0.63) was associated with lower odds of suffering a psychotic disorder compared with the *DRD2* GG/*AKT1* TT genotype (Figure 1a). A second multivariable logistic regression adjusting for the potential confounders showed a significant interaction between *DRD2/AKT1* and lifetime frequency of cannabis use on risk of psychosis (*N*=402, likelihood ratio test =11.91; *P*=0.042). Among both occasional and daily cannabis users, subjects carrying ‘risk’ allele(s) from one or both genes showed increased odds of having psychotic disorder when compared with the no ‘risk’ allele group; however, only among daily cannabis users did the increased odds of psychosis reach significance. In particular, there was a weak association between daily use and psychosis risk in subjects carrying ‘risk’ allele(s) from only one gene (OR=3.47; 95% confidence interval: 0.99, 12.13) but a strong association between daily use and psychosis risk in subjects carrying ‘risk’ allele(s) from both the genes (OR=10.06; 95% confidence interval: 1.83, 55.17). On the contrary, among those who had never used cannabis, carrying ‘risk’ allele(s) from only one (OR=0.29; 95% confidence interval: 0.08, 1.01) or both the genes (OR=0.10; 95% confidence interval: 0.02, 0.55) was associated with lower odds of suffering a psychotic disorder compared with the *DRD2* GG/*AKT1* TT genotype (Figure 1b).

## Discussion

The present results suggest an interaction between *DRD2* rs1076560 and AKT1 rs2494732 genotypes on psychosis risk among cannabis users. Individuals carrying the *DRD2* T allele or the *AKT1* C allele have an increased psychosis risk in the context of cannabis use; however, the risk is especially increased in subjects who carry ‘risk’ alleles from both genes. In line with previous findings,^1,2^ the psychosis risk in cannabis users depends on the frequency of use, with the highest probability of psychotic disorder among daily users carrying both the risk variants. Our results indicate a model of interaction known as ‘qualitative G×E interaction’ with a crossover pattern: carriers of risk allele(s) for one of the two genes (*DRD2* rs1076560 T or *AKT1* rs2494732 C allele), compared with individuals carrying no ‘risk’ alleles (*DRD2* rs1076560 GG/*AKT1* rs2494732 TT), have a lower probability of psychotic disorder if they never used cannabis but a higher probability if they have a history of cannabis use, especially of daily use. Similarly, carriers of both the ‘risk’ alleles (*DRD2* rs1076560 T allele and *AKT1* rs2494732 C allele), compared with the other groups, have the lowest probability of psychotic disorder if they never used cannabis but the highest probability if they have a history of cannabis use, especially of daily use. Our findings are in line with previous results in the field^1,2,4,5^ and indicate that specific minor alleles may prevent or promote the risk for psychosis depending on the presence and degree of cannabis use. Such findings require validation in experimental designs and animal studies where both changes in the exposure and in the genotype can be modeled.

Striatal dopamine is altered in both schizophrenia patients and cannabis users,^6^ and cannabis-induced psychosis is related to the effects of cannabis on the striatum.^7^ The *DRD2* T allele has been associated with both greater levels of striatal dopamine^8^ and cannabis-related psychosis,^2^ and is linked with AKT1 expression and psychosis-related endophenotypes by interaction with *AKT1* rs1130233.^3^ Rs1130233 is in high linkage disequilibrium with rs2494732 (*r* ^2^=0.45, *D*′=0.94) and both single-nucleotide polymorphisms have been implicated in cannabis-related psychosis,^1,7^ likely altering striatal function.^7^ Cannabinoids activate AKT1 signaling downstream of D2,^1,7^ and schizophrenia patients with comorbid substance dependence have been reported to have abnormal post-synaptic D2 function.^9^ Consistent with this, our results suggest vulnerability to the psychotogenic effects of cannabis use involving genes that control dopamine signaling, particularly postsynaptically.

## Figures and Tables

**Table 1:** Demographic measures and patterns of drug use in FEP patients and control subjects

	*FEP patients,* n*=222*	*Control subjects,* n*=228*	*Statistical comparisons*
	*M±s.d.*	*M±s.d.*	
Age of psychosis onset	26.07±6.19		
Age at assessment	26.96±6.46	28.17±7.12	F=3.54, *P*=0.06 (*ANOVA*)
Range	18–44	18–44	
			
*Distribution*			χ^*2*^ *=5.23;* P*=0.07*
18–29	151 (68.0)	145 (63.6)	
30–39	59 (26.6)	57 (25)	
40–49	12 (5.4)	26 (11.4)	
			
*Gender*			χ^*2*^, P*>0.1*
Male	149 (67.1)	141 (61.8)	
Female	73 (32.9)	87 (38.2)	
			
*Self-reported ethnicity*			χ^*2*^ *=35.44,* P*<0.001*
White Caucasian	72 (32.4)	134 (58.8)	
Black Caribbean	68 (30.6)	37 (16.2)	
Black African	57 (25.7)	31 (13.6)	
Asian/other	25 (11.3)	26 (11.4)	
			
*DRD2 rs1076560/AKT1 rs2494732*			P*>0.1*
No ‘risk’ alleles	54 (24.3)	57 (25)	
One ‘risk’ allele	124 (55.9)	133 (58.3)	
Two ‘risk’ alleles	44 (19.8)	38 (16.7)	
			
*Tobacco use*			χ^*2*^ *=27.19,* P*<0.001*
Nicotine dependence	157 (70.7)	106 (46.5)	
Not nicotine dependence	65 (29.3)	122 (53.5)	
			
*Stimulant use*			P*>0.1*
Yes	93 (41.9)	81 (35.5)	
Never	129 (58.1)	147 (64.5)	
			
*Alcohol use*			χ^*2*^ *=23.58,* P*<0.001*
Harmful drinking behavior	157 (70.7)	203 (89)	
Not harmful drinking behavior	65 (29.3)	25 (11)	
			
*Cannabis use*			χ^*2*^ *=4.00,* P*=0.05*
Yes	158 (71.2)	142 (62.3)	
No ‘risk’ alleles	40 (25.3)	36 (25.4)	
One ‘risk’ allele	89 (56.3)	82 (57.7)	
Two ‘risk’ alleles	29 (18.3)	24 (23.2)	
Never	64 (28.8)	86 (37.7)	
No ‘risk’ alleles	14 (21.9)	21 (24.4)	
One ‘risk’ allele	35 (54.7)	51 (59.3)	
Two ‘risk’ alleles	15 (23.4)	14 (16.3)	
			
*Frequency of use*			χ^*2*^ *=14.38,* P*<0.001*
Daily^a^	92 (61.1)	43 (39.8)	
No ‘risk’ alleles	24 (26.1)	11 (25.6)	
One ‘risk’ allele	51 (55.4)	22 (51.2)	
Two ‘risk’ alleles	17 (18.5)	10 (23.2)	
Weekly or less^a^	52 (38.9)	65 (60.2)	
No ‘risk’ alleles	12 (23.1)	18 (27.7)	
One ‘risk’ allele	29 (55.7)	37 (56.9)	
Two ‘risk’ alleles	11 (21.2)	10 (15.4)	
No details^a^	14	34	
			
*Age of first use*^a^	*16.51±4.99*	*16.51±5.81*	*P>0.1 (ANOVA)*
No ‘risk’ alleles	16.35±4.63	16.20±7.46	
One ‘risk’ allele	16.51±5.03	16.64±5.10	
Two ‘risk’ alleles	16.66±5.35	16.6±4.83	
Age of onset minus age of first use^b^	9.56±7.00		

Abbreviations: ANOVA, analysis of variance; FEP, first-episode psychosis; no ‘risk’ alleles, *DRD2* GG/*AKT1* TT; one ‘risk’ allele, *DRD2* T carriers/*AKT1* TT+*DRD2* GG/*AKT1* C carriers; two ‘risk’ alleles, *DRD2* T carriers/*AKT1* C carriers.

a In those who had ever used cannabis.

b No patient started to use cannabis for the first time after the psychosis onset.

Data are presented as M±s.d. or *n* (%).
